# Eco-Friendly Algicidal Potential of *Zanthoxylum bungeanum* Leaf Extracts: Extraction Optimization and Impact on Algal Growth

**DOI:** 10.3390/microorganisms13040760

**Published:** 2025-03-27

**Authors:** Jie Cheng, Long Tan, Yaxin Han, Mengya Hou, Zhenxia Zhu, Xiu Zhang, Qing Guo, Kaidian Zhang, Jiashun Li, Yang Zhang, Chaobo Zhang

**Affiliations:** 1 State Key Laboratory of Macromolecular Drugs and Large-Scale Preparation, School of Pharmaceutical Sciences and Food Engineering, Liaocheng University, Liaocheng 252000, China; chengjie@lcu.edu.cn (J.C.);; 2 State Key Laboratory of Marine Resource Utilization in the South China Sea, Hainan University, Haikou 570100, China; kzhang@hainanu.edu.cn

**Keywords:** *Z. bungeanum* leaf, natural flavonoids, response surface methodology, biological control, eco-friendly algicide, *Tetrodesmus obliquus*

## Abstract

*Zanthoxylum bungeanum* leaves were regarded as a waste byproduct for a long period of time, yet their functional components presented potential as novel antimicrobial agents. However, their effectiveness in controlling algal blooms remains unexplored. In this study, the inhibition effect of *Z. bungeanum* leaf extracts on algal blooms was firstly demonstrated, and the flavonoid profiles of the leaf extract were identified using non-targeted metabolomics analysis. Then, response surface methodology was performed for extraction to further evaluate the feasibility of industrial application. Specifically, the effects of extracts on the cell density, photosynthetic efficiency, and antioxidant activity of *Tetrodesmus obliquus* was investigated. The results showed that the extraction yield of flavonoids from *Z. bungeanum* leaves reached 6.85% under the optimized conditions of an ultrasonic power of 600 W, an LSR of 20:1 mL/g, an ethanol concentration of 77.5%, an ultrasonic duration of 18 min, and an ultrasonic temperature of 80 °C, which significantly decreased the Fv/Fm and PIabs values by 54.60% and 98.22%, respectively, after exposure of *T. obliquus* to 40.0 mg/L *Z. bungeanum* leaf extract for 66 h. Meanwhile, treatment with *Z. bungeanum* leaf extract at a dose of 40.0 mg/L generated T-AOC values that were 4.0 times higher than the control without the addition of *Z. bungeanum* leaf extracts. These results suggest that *Z. bungeanum* leaf extracts could be used in the development of potentially effective biological algicides. Our study provides data to support the development of algicides and realizes the resource application of *Z. bungeanum* leaf waste, achieving a synergistic outcome of both economic and ecological benefits.

## 1. Introduction

*Zanthoxylum bungeanum*, a native shrub belonging to the Rutaceae family, is the predominant species extensively cultivated in China, and its annual output exceeds 500,000 tons [1,2]. China has a long tradition of cultivating and breeding *Z. bungeanum*, resulting in a rich repository of germplasm resources [3]. A growing body of evidence has demonstrated the diverse bioactivities of *Z. bungeanum*, including antioxidant, antitumor, anti-inflammatory, antimicrobial, and insecticidal properties [4,5,6]. Furthermore, previous studies have identified numerous compounds in *Z. bungeanum*, with amides, essential oils, and flavonoids being reported as the major active constituents [2,7]. To date, research on *Z. bungeanum* has mainly focused on the isolation and identification of active ingredients in the fruit of *Z. bungeanum*. Increasing efforts have also been made to study the pharmacological and toxicological evaluation of active constituents from the fruit of *Z. bungeanum* [8].

As a byproduct, *Z. bungeanum* leaves were generally ignored for a long period of time. However, they showed similar functional components to *Z. bungeanum*. Accordingly, *Z. bungeanum* leaves may have provided an opportunity to create further financial benefits. Recent research has mainly focused on the flavonoid compounds present in *Z. bungeanum* leaves, which are widely used in nutrition, food, and medicine due to their significant biological roles in human health [3]. Previous investigations have shown that *Z. bungeanum* leaves contain antioxidant flavonoids that are effective in scavenging free radicals [6,9], and several investigations have been conducted on the yield of flavonoids in *Z. bungeanum* leaves under both biotic and abiotic stress conditions [10,11,12,13]. Nonetheless, the antimicrobial properties of flavonoid components from *Z. bungeanum* leaves in controlling algal blooms have been relatively understudied.

Flavonoids are ubiquitous low-molecular-weight phenolic compounds synthesized by plants and have a broad spectrum of well-established antioxidative, anti-inflammatory, antimutagenic, anticarcinogenic, and antibacterial properties [14,15,16]. Traditional solvent extraction techniques for flavonoids in the laboratory, such as Mceration and Soxhlet extraction, are time consuming and require high solvent consumption. Several modern approaches, including ultrasonic and microwave-assisted extraction as well as supercritical fluid extraction, are attracting increasing interest due to their high yield and low cost [17,18]. Recent developments in ultrasonic extraction technology enable the extraction of active ingredients from plants into a solvent using vibrations, cavitation, and comminution generated using ultrasonic technology [19]. Various factors have been shown to affect the extraction process, such as ultrasonic power, extraction solvent, extraction time, extraction temperature, and the liquid-to-solid ratio (LSR) [20,21]. In this case, response surface methodology (RSM) has emerged as a common optimization technique. When appropriately designed, RSM constructs a binary regression equation model using a given set of experimental data. Ensuring the accuracy and reliability of the model, it effectively predicts an optimal combination of parameters to achieve the best experimental results. During the optimization process, RSM analyzes all levels of test factors, thereby overcoming the limitations of previous orthogonal experiments, which were limited to the analysis of isolated points but could not provide intuitive graphics [22,23].

Algal blooms are defined as the rapid accumulation of algal biomass in aquatic ecosystems and the release of toxins and harmful metabolites by some algal species [24]. Algal blooms occur frequently in eutrophic water [25] and are becoming more frequent, intense, and geographically varied in distribution [26]. In addition to being a growing environmental problem, harmful algal blooms have serious impacts on aquatic organisms, the aquaculture industry, and public health worldwide [27]. The destructive proliferation of algal blooms has become a major global concern associated with the degradation of aquatic ecosystems. Currently, the primary techniques to control algal blooms include physical and chemical approaches with the disadvantages of high cost and being environmentally unfriendly [28]. Inspired by natural phenomena, environmentally friendly algicidal methods using allelopathic substances offer the advantages of convenient application and low costs, thus offering a promising application prospect in the fields of water and ecological restoration [29]. One notable study investigated the algicidal properties of extracts from the invasive plant *Arundo donax* against the harmful alga *Prymnesium parvum* [30]. This research demonstrated that the plant extract exhibited both growth-suppressing and algicidal activities. Gramine, a constituent of the extract, was particularly effective, showing partial algicidal and algistatic activity, suggesting that *Arundo donax* could be a valuable source of natural products for controlling algal blooms. Similarly, the potential of *Spartina alterniflora* was explored for its ability to inhibit the growth of harmful algae such as *Phaeocystis globosa* and *Prorocentrum donghaiense* [31]. This study found that high concentrations of *S. alterniflora* extracts significantly reduced the growth of these algal species, indicating its potential as a novel antialgal agent. The presence of flavonoids in the extracts, known for their antialgal properties, further supports the use of *S. alterniflora* in managing HABs. These studies underscore the potential of plant extracts as natural algicides, offering a sustainable and eco-friendly solution to the problem of harmful algal blooms. Further research into the specific mechanisms and efficacy of these natural compounds will be crucial in developing effective strategies for algal bloom management. Moreover, the mode of action by which plant-derived allelopathic substances exhibit anti-algal effects primarily involves cellular structure damage and interference with essential biological functions, including oxidative stress responses, triggering programmed cell death pathways, impairing photosynthetic efficiency, and disrupting protein biosynthesis processes [32]. To our knowledge, there is a limited number of studies on the allelopathic mechanism of *Z. bungeanum* leaf extracts.

To date, a variety of algae species are capable of rapid reproduction and can lead to the formation of algal blooms when conditions are favorable. The dominant species in these blooms change with the seasons. Specifically, green algae typically prevail in spring and autumn, while cyanobacteria are more dominant in summer [33]. *Microcystis aeruginosa* is the most prevalent cyanobacterial species responsible for algal blooms, and the use of allelochemicals has been demonstrated to be highly effective in controlling its growth [34]. *Tetrodesmus obliquus* is a common green algal species that often co-exists with cyanobacteria [35] and involves in the formation of mixed blooms [36]. During periods of eutrophication, *T. obliquus* may thrive alongside harmful cyanobacteria, leading to shifts in community composition and potential impacts on water quality and biodiversity [36]. However, limited studies focus on the use of allelochemicals to inhibit the growth of *T. obliquus*.

In this study, the effect of *Z. bungeanum* leaf extracts on algal blooms control was first evaluated. Then, the ultrasonic-assisted extraction process to obtain total flavonoids (TFs) from *Z. bungeanum* leaves was optimized using response surface methodology. We suggested that the use of the allelopathic application showed potential as an effective approach to manage detrimental algal blooms. Meanwhile, allelochemicals induced damage at multiple levels of physiological and biochemical processes in *T. obliquus*, including reductions in algal cell density, interference with photosynthesis, and activation of the antioxidant system. This study explores, for the first time, the use of *Z. bungeanum* leaf extracts as a biological algicide and investigates its impact on algal physiology. Unlike previous studies focusing on synthetic or non-targeted plant-derived compounds, we employ metabolomic profiling to identify bioactive flavonoids and optimize the extraction process for sustainable application. This study provides data to support the development of algicides and realizes the resource application of *Z. bungeanum* leaf waste, achieving a synergistic outcome of both economic and ecological benefits.

## 2. Materials and Methods

### 2.1. Experimental Materials and Reagents

The green algae used, *T. obliquus* (FACHB-417), was commonly observed in freshwater settings, obtained from the Freshwater Algae Culture Collection of the Institute of Hydrobiology of the Chinese Academy of Sciences, and cultured in liquid BG-11 media [37]. Cultures were grown in autoclaved 250 mL Erlenmeyer flasks containing 50 mL of fresh medium at a temperature of 25 ± 1 °C. The cells were then transferred to an illumination incubator that operated on 14 h/10 h light/dark cycle with rotational shaking (at 120 rpm) to avoid algae sedimentation. The cultures were maintained at a light intensity of 6000 lux, and the incubation period was terminated after approximately 66 h.

*Z. bungeanum* leaves were acquired from Chongqing Fuliang Grain and Oil Co., Ltd., (Chongqing), China, a company authorized to harvest *Z. bungeanum*. Furthermore, the *Z. bungeanum* purchase adhered to pertinent institutional, national, and international rules and regulations. Rutin was acquired from Aladdin Holdings Group Co., Ltd. (Beijing, China). Ethanol (64-17-5), aluminum nitrate (7784-27-2), sodium nitrite (7632-00-0) and sodium hydroxide (1310-73-2) were acquired from Sinopharm Chemical Reagent Co., Ltd. (Shanghai, China). All further reagents employed were of analytical grade.

### 2.2. Ultrasound-Assisted Extraction of Flavonoids from Z. bungeanum Leaves

The dried *Z. bungeanum* leaves obtained were pulverized with a grinder and then filtered through 60-mesh, 80-mesh, and 140-mesh sieves. They were then packed and dry-kept for future use. A 0.1 g specimen of dehydrated *Z. bungeanum* leaf powder was placed in a 5.0 mL centrifuge tube, followed by the addition of several solvents. The mixture was then introduced into the thermostatic ultrasonic cleaner (JP-100S, Skymen Cleaning Equipment Shenzhen Co., Ltd., Shenzhen, China), which was set to a constant ultrasound power of 300 W or 600 W and an ultrasonic frequency of 40 kHz.

Meanwhile, the temperature of the mixture’s temperature was controlled using the digital temperature display on the thermostatic ultrasonic cleaner. Different conditions were used to establish distinct values for the LSR, extraction duration, ethanol volume percent, and extraction temperature. Upon completion of the extraction, the extraction vessels were allowed to cool to ambient temperature. TF analysis was performed on the supernatant obtained by centrifuging the mixture at 12,000 rpm for 5 min. A rotary evaporator (SY-2000, Shanghai Yarong Biochemical Instrument Factory, Shanghai, China) was used to enhance the TF content.

### 2.3. Determination of Flavonoids from Z. bungeanum Leaves

Quantification of TFs was conducted using the aluminum nitrate colorimetric technique with certain adjustments [38]. Typically, 0.2 mL of an extracted solution containing flavonoids was added to a 5.0 mL volumetric flask. A mixture of 1.8 mL of 80% (*v*/*v*) ethanol and 0.12 mL of a 5% sodium nitrite solution was combined for 6 min. Then, 0.12 mL of a 10% solution of Al(NO_3_)_3_ (*w*/*v*) was added and well mixed. After an interval of 6 min, 1.6 mL of a 4% (*w*/*v*) NaOH solution and 0.16 mL of deionized water were introduced to reach the target volume. The absorbance of the test solution at 510 nm was measured with a spectrophotometer after a 15 min incubation at 25 °C.

A calibration curve was generated by preparing rutin solutions in 60% ethanol with concentrations spanning from 0.00 to 0.25 mg/mL, and a rutin standard curve was plotted with the mass concentration of its standard sample as the X-axis and the measured absorbance as the Y-axis. Based on the curve plotted, a corresponding linear regression curve of rutin concentration and absorbance value was obtained. A regression equation, Y = 10.755X + 0.0619, was estimated from the regression curve. A strong linear association was found throughout the range of 0.00–0.25 mg/mL with a correlation coefficient R^2^ of 0.9957 (Figure S1). The regression equation was used to determine TF levels in extracts. The TF yield was calculated as Y = (C × V)/W × 100%, where C shows the extract concentration (mg/mL), V denotes the extract volume (mL), and W denotes the specimen mass (g).

### 2.4. Metabolomics Determination of Flavonoids from Z. bungeanum Leaves

Analyses of *Z. bungeanum* leaf extracts were carried out using ultra-high-performance liquid chromatography (LC-30A, Shimadzu, Kyoto, Japan) and high-resolution mass spectrometry (AB TripleTOF 6600, Sciex, Framingham, MA, USA). The LC-MS/MS system was operated under the following conditions: HPLC column, Waters ACQUITY UPLC HSS T3 (100 × 2.1 mm, 1.8 µm, Waters Corporation, Milford, MA, USA); column temperature, 40 °C; flow rate, 0.4 mL/min; injection volume, 4 µL; solvent system, water (0.1% formic acid):acetonitrile (0.1% formic acid); gradient program, 95:5 *v*/*v* at 0 min, 35:65 *v*/*v* at 5.0 min, 1:99 *v*/*v* at 6.0 min, 1:99 *v*/*v* at 7.5 min, 95:5 *v*/*v* at 7.6 min, and 95:5 *v*/*v* at 10.0 min. The data acquisition was operated using the information-dependent acquisition (IDA) mode using Analyst TF 1.7.1 Software (Sciex, Concord, ON, Canada). The source parameters were set as follows: ion source gas 1 (GAS1), 50 psi; ion source gas 2 (GAS2), 60 psi; curtain gas (CUR), 35 psi; temperature (TEM), 550 °C; declustering potential (DP), 80 V or −80 V for positive or negative modes, respectively; and ion spray voltage floating (ISVF), 5500 V or −4500 V for positive or negative modes, respectively. The TOF MS scan parameters were set as follows: mass range, 50–1250 Da; accumulation time, 200 ms; and dynamic background subtract, on. The product ion scan parameters were set as follows: mass range, 50–1250 Da; accumulation time, 40 ms; collision energy, 30 or −30 V for positive or negative modes, respectively; collision energy spread, 15; resolution, UNIT; charge state, 1 to 1; intensity, 100 cps; exclude isotopes within 4 Da; mass tolerance, 50 mDa; maximum number of candidate ions to monitor per cycle, 12.

### 2.5. Optimization of Ultrasonic-Assisted Extraction of Total Flavonoids from Z. bungeanum Leaves

As each factor was examined, all other parameters were held constant. The single-factor assay was conducted under the following levels: LSRs (5:1, 10:1, 15:1, 20:1, and 25:1 mL/g); extraction time (10, 20, 30, 40, and 50 min); ethanol volume fractions (20%, 40%, 60%, 80%, and 100%); and extraction temperatures (40, 50, 60, 70, and 80 °C). Based on the single-factor experiments, the independent variables that significantly influenced the extraction process were screened, including LSRs, extraction time, and ethanol volume fractions. A three-factor, three-level Box–Behnken design (BBD) was used, with each variable adjusted at three different levels (Table S1) to maximize the production of TFs. Typically, 17 experiments were run in a randomized sequence, with the flavonoid yield as the response value in order to minimize the impact of uncontrolled variables. The data were analyzed with a second-order polynomial regression model. The variable with the greatest impact was identified from response surface plots and contour plots generated by the 3D model using Design-Expert 8.0.6 software (Stat-Ease, Inc., Minneapolis, MN, USA) (Table 1).

### 2.6. Microalgae Growth and Photosynthetic Inhibition Assay

*Z. bungeanum* leaf extracts were obtained using optimal process parameters, concentrated using a rotary evaporator to improve the flavonoids content, and introduced into cultures containing exponentially growing algae to achieve final gradient concentrations of 0, 20, and 40 mg/L. Cell cultures were grown in autoclaved 250 mL Erlenmeyer flasks containing 50 mL of BG-11 media at a temperature of 25 ± 1 °C. The experimental settings were identical to those outlined in Section 2.1. The cell density of *T. obliquus* was assessed using microscopic photography. Chlorophyll a fluorescence transient was determined using a chlorophyll fluorometer (AquaPen-C AP110-C, Photon Systems Instruments, Drásov, Czech Republic), and the JIP transients were quantified following exposure to *Z. bungeanum* leaf extracts. Before measurement, 3.0 mL microalgae cultures treated with *Z. bungeanum* leaf extract were centrifuged at 8000 rpm for 5 min. Then, fresh BG-11 medium was used to resuspend these algal cells to ensure that the OD_680_ of each treatment group was at the same level. Before measuring the fluorescence parameter at different time intervals (1, 3, 18, 42, and 66 h), the test samples were preconditioned in a dark atmosphere for a minimum of 20 min.

### 2.7. Measurement of the Total Antioxidant Activity

The possible oxidative stress generated by *Z. bungeanum* leaf extracts was determined by performing an oxidative stress assessment using the Total Antioxidant Activity Kit (Nanjing Jiancheng Bioengineering Institute, Nanjing, China) to determine free radical levels [39]. Briefly, 4.0 mL of the test microalgae cells were centrifuged at 8000 rpm for 5 min, and the supernatant was discarded. The precipitate was reconstituted in 0.5 mL of BugBuster^®^ Protein Extraction Reagent and allowed to absorb for 30 min. After centrifugation at 12,000 rpm for 10 min, the supernatant was collected for the quantification of protein content and total antioxidant activity. Protein content and total antioxidant activity were determined with assay kits (Nanjing Jiancheng Bioengineering Institute, Nanjing, China) in accordance with the manufacturer’s protocols. Each unit of total antioxidant capacity (U) was expressed as the absorbance value (OD_520_) of the reaction system increasing by 0.01 per milliliter of algal cells per minute. The total antioxidant activity data were expressed as units per milligram of total soluble proteins (U/mg·protein).

### 2.8. Data Analysis

All data are expressed as means ± standard deviation (SD, *n* = 3). Univariate analysis of variance (ANOVA) was performed on the data obtained, followed by Duncan’s multiple comparative test. A 5% significance level was set as statistical significance (*p* < 0.05). Experimental data were processed using SPSS 19.0 statistical software (SPSS Inc., Chicago, IL, USA), and diagram plotting was generated using the Origin 2021 software (San Diego, CA, USA).

## 3. Results

### 3.1. Z. bungeanum Leaf Extracts Offer a Promising Application Prospect to Safely Control the Outbreak of Algal Blooms and the Identification of the Flavonoid Profile of Z. bungeanum Leaves

The leaves of *Z. bungeanum* possess functional components analogous to those of *Z. bungeanum*, with flavonoids reported as the primary active constituents. Flavonoids have a broad spectrum of established antioxidative, anti-inflammatory, antimutagenic, anticarcinogenic, and antibacterial properties. This suggests that *Z. bungeanum* leaf extracts could be used in the development of potentially effective biological algicides.

**Figure 1:**
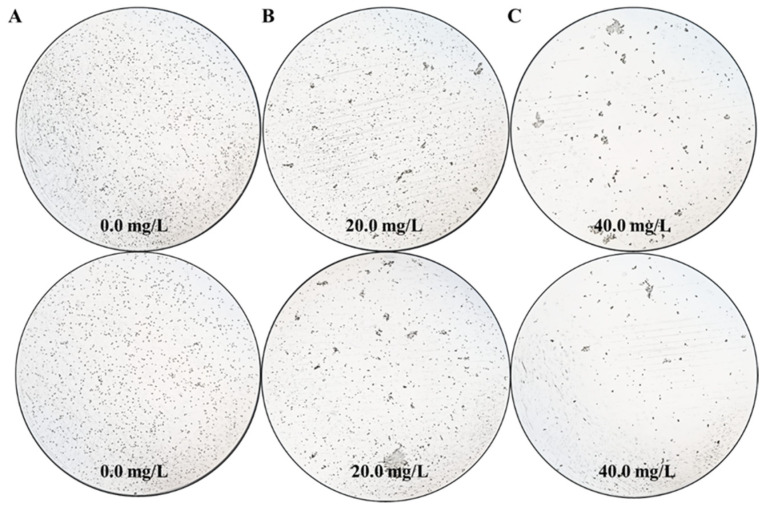
The effects of exposure time (66 h) with different *Z. bungeanum* leaf extract treatments on the cell density of *T. obliquus*. Significant differences between treatments for each *Z. bungeanum* leaf extract concentrations were noted at 0.0 mg/L (**A**), 20.0 mg/L (**B**), and 40.0 mg/L (**C**).

As a proof of concept, the effects of different *Z. bungeanum* leaf extract treatments on the cell density of *T. obliquus* are shown in Figure 1. Microscopic analysis revealed that treatments with different concentrations of *Z. bungeanum* leaf extract exhibited significant concentration-dependent inhibition of *T. obliquus* growth. After 66 h of exposure, a low concentration (20.0 mg/L TF) of *Z. bungeanum* leaf extracts slightly inhibited the cell density of *T. obliquus*. In contrast, the cell density in the high concentration (40.0 mg/L TF) treatment group decreased to the lowest levels observed, indicating that the higher concentration exerted a more pronounced inhibitory effect. Notably, when treated with *Z. bungeanum* leaf extracts, the cells appeared uneven and aggregated together in certain dense areas, which may represent a maladaptive strategy for coping with adversity stress. These results demonstrated that the cell density of *T. obliquus* can be inhibited by the flavonoid components present in *Z. bungeanum* leaves. This strategy shows potential advantages over previous techniques to control algal blooms using physical and chemical approaches, which have the disadvantages of being costly and environmentally unfriendly.

**Figure 2:**
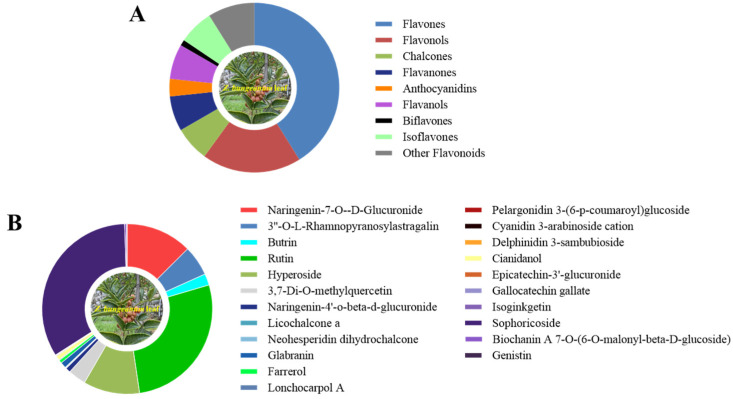
The flavonoid compounds detected in the leaf extracts of *Z. bungeanum* using non-targeted metabolomics analysis. (**A**) The subclass of flavonoid compounds in *Z. bungeanum* leaf extract, (**B**) the top three individual flavonoid compounds from each subclass in the leaf extracts.

Non-targeted metabolomics analysis of the *Z. bungeanum* leaf extract was further carried out to identify the flavonoid profile present in it (Figure 2). The results revealed the detection of a total of 90 flavonoid compounds in the *Z. bungeanum* leaf extract that belong to nine subclass: flavones, flavonols, chalcones, flavanones, anthocyanidins, flavanols, biflavones, isoflavones, and other flavonoids (Figure 2A). The relative abundances of the individual flavonoid compounds in the *Z. bungeanum* leaf extract were recorded in Table S2. The pie chart in Figure 2B presents the relative abundances of the top three individual flavonoid compounds from each subclass in the *Z. bungeanum* leaf extract. The identified flavones include naringenin-7-O-D-glucuronide, 3′-O-L-rhamnopyranosylastragalin, and butrin. In the flavonols class, seventeen compounds were identified, among which rutin, hyperoside, and 3,7-di-O-methylquercetin were the most abundant compounds. Furthermore, in the chalcones class, six compounds were identified, including naringenin-4′-O-beta-D-glucuronide, licochalcone a, neohesperidin dihydrochalcone, xanthohumol, flavokawain b, and isobavachalcone. Also, six compounds were identified in flavanone, flavanol, and isoflavone classes. The identified anthocyanidins include pelargonidin 3-(6-p-coumaroyl)glucoside, cyanidin 3-arabinoside cation, and delphinidin 3-sambubioside. Only one compound was identified in biflavones class. It is worth noting that the relative content of sophoricoside and rutin was the highest among the flavonoid compounds in the *Z. bungeanum* leaf extracts (Table S2). These results suggest that allelopathic flavonoids were abundant in the *Z. bungeanum* leaf extracts, and they were involved in the inhibition of the growth of *T. obliquus*.

### 3.2. Optimized the Extraction Technique of TFs

#### 3.2.1. Single Factor Experiment Results

We investigated the effects of various factors on the extraction process of TFs from *Z. bungeanum* leaves using single-factor experiments, including ultrasonic power, LSR, ethanol concentration, ultrasonic time, and ultrasonic temperature. The dried *Z. bungeanum* leaves were pulverized with a grinder and sieved through 60-mesh (sample 1), 80-mesh (sample 2), and 140-mesh (sample 3) sieves. The different solvents were added to the dried *Z. bungeanum* leaf powder, and the mixture was then placed in the thermostatic ultrasonic cleaner equipped with a fixed ultrasound power. We first investigated the effect of ultrasonic power on the efficiency of flavonoid extraction from *Z. bungeanum* leaves. The yield of flavonoids was enhanced by increasing ultrasound power in all samples (Figure S2). Specifically, compared to ultrasound power of 300 W, the yield of flavonoids under 600 W ultrasound power increased by 2.77%, 12.46%, and 5.32% in sample 1, sample 2, and sample 3, respectively, indicating that the treatment with 600 W ultrasound power was more conducive to the dissolution of flavonoids entering the extraction solvent from *Z. bungeanum* leaf powder. Consequently, 600 W ultrasound power was chosen for additional experiments.

##### Effects of the Liquid-to-Solid Ratio on Flavonoid Extraction Performance

**Figure 3:**
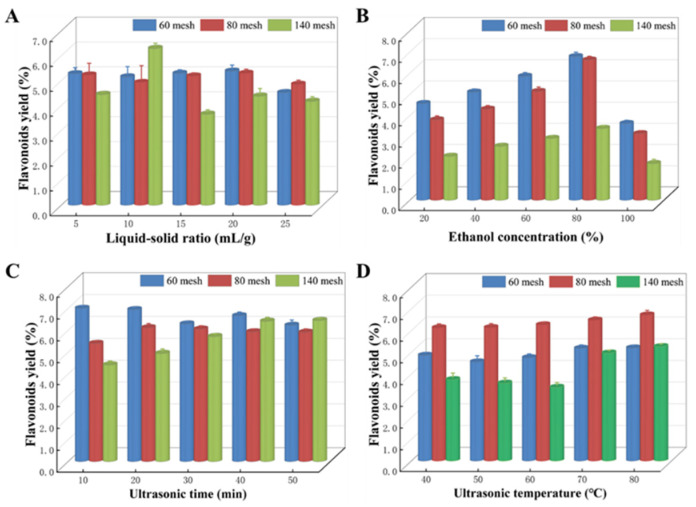
Various factors exert an impact on the extraction process in different samples. (**A**) Liquid-to-solid ratio, (**B**) ethanol concentration, (**C**) ultrasonic time, and (**D**) ultrasonic temperature. Values represent the mean of three independent measurements (*n* = 3), and bars indicate SDs. All processes were biologically repeated in three independent and parallel experiments.

We maintained a constant ethanol concentration of 60%, ultrasound temperature of 60 °C, and ultrasound duration of 20 min to investigate the impact of various LSRs (5:1, 10:1, 15:1, 20:1, and 25:1 mL/g) on the extraction efficiency of *Z. bungeanum* leaves. The results obtained are shown in Figure 3A. The findings indicated that the production of flavonoids from *Z. bungeanum* leaves in sample 1 and sample 2 exhibited a progressive increase with increasing LSRs. However, a decrease in flavonoid yield was observed when the LSR exceeded 20:1 mL/g. However, the flavonoid yield of *Z. bungeanum* leaves in sample 3 peaked at 10:1 mL/g.

##### Effects of Ethanol Concentration on Flavonoid Performance

The effect of ethanol concentration on total flavonoid production is shown in Figure 3B. Ethanol concentrations of 20%, 40%, 60%, 80%, and 100% were used, and the procedure was carried out at 60 °C for 20 min. The extraction yield exhibited a significant increase as the ethanol concentration increased from 20% to 80%, but then decreased as the ethanol concentration increased. TF extraction yield reached a maximum of 80% in all samples. Hence, an ethanol concentration of 80% was chosen as the center point for the RSM assay.

##### Effects of Ultrasonic Time on Flavonoid Performance

Different ultrasonic times (10, 20, 30, 40, and 50 min) were used to study the extraction performance of flavonoids (Figure 3C). The ultrasound time had significant variations in the yield of flavonoids among samples. Specifically, the flavonoid yield in sample 1 showed a decreasing trend within the set time range when the sonication time was prolonged. However, the opposite trend was observed in sample 3. In addition, flavonoid production in sample 2 exhibited an increase as the duration of ultrasonic exposure was increased. After an extraction time of 20 min, the yield of flavonoids peaked and then gradually decreased.

##### Effects of Ultrasonic Temperature on Flavonoid Performance

Using the single factor approach, we investigated the effect of five different levels (40, 50, 60, 70, and 80 °C) on the rate of flavonoid extraction while keeping all other extraction parameters constant (Figure 3D). The extraction yield in all test samples increased steadily with the increasing temperature, reaching its peak at 80 °C. Thus, the ideal temperature was determined to be 80 °C.

According to the results of the above single factor tests, the optimum flavonoid yields in samples 1, samples 2, and samples 3 were 5.204%, 6.720%, and 5.269%, respectively (Table S3). As shown in Table S3, the ultrasonic-assisted extraction efficiency of TFs from *Z. bungeanum* leaves was significantly affected by sieve treatment with different pore sizes, and the maximum flavonoid content was obtained in sample 2. As a result, sample 2 was used for further optimization of the extraction process of TFs using RSM.

#### 3.2.2. Response Surface Analysis

##### Model Fitting

Based on the previous single-factor experiments, three variables that significantly influenced the extraction process were chosen to optimize the yield of TFs using a three-factor, three-level BBD (Table S1). The yield of flavonoids was used as the response value, and 17 experiments were conducted using a BBD. The flavonoid yield ranged from 1.397 to 6.989, and the highest flavonoid production was achieved with an LSR of 20:1 mL/g, ultrasonic interval of 20 min, and ethanol concentration of 80% (Table 1). Simultaneously, the experimental data of the BBD were fitted to a second-order polynomial model established by multiple regression analysis. The equation extracted from the BBD experiment was as follows: Y = 6.73 − 0.7017A − 0.4006B − 0.0071C + 0.2203AB − 0.3983AC + 0.2550BC –2.94A^2^ − 1.16B^2^ − 0.1981C^2^, where A, B, and C were the ethanol concentration, ultrasonic time, and the LSR, respectively.

##### Response Surface Plots and Contour Plot Analysis

**Figure 4:**
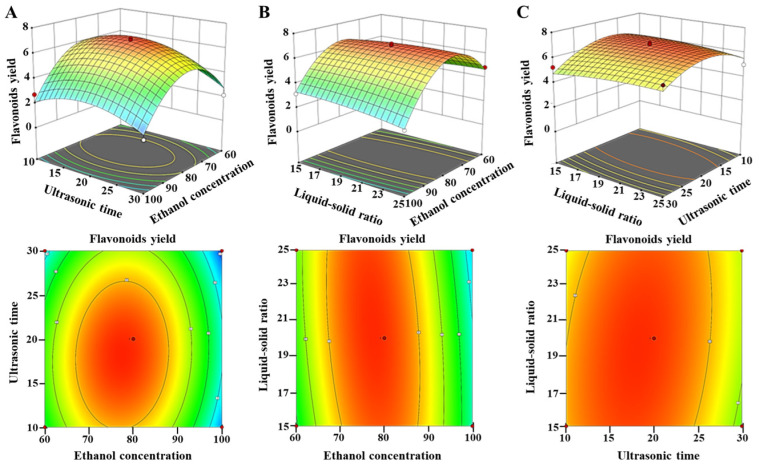
The three-dimensional response surface plots and two-dimensional contour plots showing the interaction effects between various factors affecting flavonoid yield. (**A**) The effects of ethanol concentration, ultrasonic time, and their interaction on the yield of flavonoids; (**B**) the effects of ethanol concentration, liquid-to-solid ratio, and their interaction on the yield of flavonoids; (**C**) The effects of ultrasonic time, liquid-to-solid ratio, and their interaction on the yield of flavonoids.

Figure 4 shows the graphic representations of the response surface and contour plots according to the regression equation in order to further analyze and evaluate the interaction effects between various factors affecting flavonoid yield. The effect of ethanol content, ultrasonic duration, and their interaction on the production of flavonoids is shown (Figure 4A). The effect of ethanol concentration on the flavonoid output was found to be significant, whereas the influence of ultrasonic duration on the flavonoid yield was comparatively less pronounced. Specifically, a lower ethanol concentration or ultrasonic time produced a lower yield. The flavonoid yield reached a maximum when the ethanol concentration was 77.50% and the ultrasonic time was 18.12 min. Then, the flavonoid yield declined after 77.50% and 18.12 min (Figure S3). At the same time, the contours suggested that the relationship between the ethanol content and ultrasonic duration was not statistically significant, which aligns with the findings shown in Table 2.

Figure 4B illustrates the effect of the LSR, ethanol concentration, and their interaction on the production of flavonoids. The flavonoid production exhibited an upward trend as the ethanol concentration increased in the range of 60–77.50%, and the LSR increased within the range of 15.0–20.0 mL/g (Figure S3). The maximum flavonoid production was achieved at an LSR of 20.0 mL/g and an ethanol concentration of 77.50%. Nevertheless, the productivity decreased when the ethanol content was above 77.50%, and the LSR surpassed 20.0 mL/g. These results suggested that increasing the LSR and ethanol concentration seemed to have a negative effect on the extract yield. Furthermore, the impact of the LSR on flavonoid production was shown to be less significant when compared to the ethanol concentration. Additionally, the interaction effect between the ethanol concentration and LSR was not statistically significant.

Figure 4C illustrates the effect of the LSR, ultrasonic temporal duration, and their interaction on the production of flavonoids. The extraction of flavonoids showed a positive correlation with the increasing LSR and ultrasonic duration. The maximum flavonoid yield was obtained when the LSR and ultrasonic duration were set at 20.0 mL/g for 18.1 min (Figure S3). However, the mutual interactions between the LSR and ultrasound duration were not statistically significant due to the circular shape of the contour plot.

By analyzing the response surface data, the optimal parameters for achieving the highest flavonoid production were determined using an overall evaluation. These parameters include an ultrasonic power of 600 W, an LSR of 20:1 mL/g, an ethanol concentration of 77.5%, an ultrasonic duration of 18.12 min, and an ultrasonic temperature of 80 °C. TFs from *Z. bungeanum* leaf were extracted to facilitate studies using the following precise parameters: ultrasonic power of 600 W, LSR of 20:1 mL/g, ethanol concentration of 77.5%, ultrasonic duration of 18.0 min, and ultrasonic temperature of 80 °C. To validate the accuracy of the response model, a verification experiment was conducted under the aforementioned optimal conditions. The experimental results showed that the flavonoid extraction yield from *Z. bungeanum* leaves achieved 6.850%, while the model-predicted value was 6.812%. This close alignment between experimental data and theoretical predictions confirms the model’s reliability in forecasting extraction outcomes, demonstrating its effectiveness in response prediction. Consequently, *Z. bungeanum* leaf extracts were prepared under these conditions for further toxicological evaluations, and the total flavonoid content was enhanced using a rotary evaporator.

### 3.3. Inhibition of Photosynthetic Activity of T. obliquus by Z. bungeanum Leaf Extracts

Chlorophyll fluorescence induction kinetic curves were employed to investigate the effects of *Z. bungeanum* leaf extract treatments on the photosynthetic activity of *T. obliquus* (Figure S4). As expected, all groups revealed a distinct polyphasic increase in the fluorescence induction (O-J-I-P) curves when exposed to different doses of *Z. bungeanum* leaf extract treatments, and the shapes of the fluorescence induction curves exhibited more pronounced alterations across different concentration gradients of *Z. bungeanum* leaf extracts. In particular, fluorescence yields at the J and I phases reported a significant elevation (*p* < 0.05) with escalating dosages of *Z. bungeanum* leaf extracts during the first 18 h. Conversely, a marked decline in fluorescence yield was observed after 66 h of exposure, suggesting that the photosynthetic apparatus may respond to *Z. bungeanum* leaf extracts by closing the photosystem II (PSII) reaction center.

**Figure 5:**
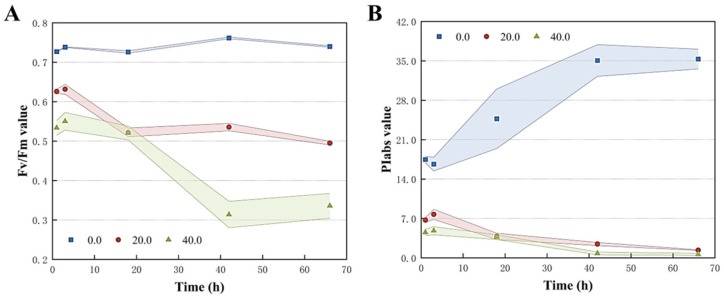
Variations of Fv/Fm (**A**) and PIabs (**B**) of *T. obliquus* in response to exposure time to different *Z. bungeanum* leaf extract concentrations. Significant differences between treatments for each *Z. bungeanum* leaf extract concentration was observed. Values represent the mean of three independent measurements (*n* = 3), and shaded error margins indicate SDs. All processes were biologically repeated in three independent and parallel experiments.

The photosynthetic efficiency of *T. obliquus* treated with *Z. bungeanum* leaf extracts was analyzed using the JIP test. The characteristics of the fluorescence induction kinetic curves of *T. obliquus* at various doses of *Z. bungeanum* leaf extracts were compared. As an indicator of PSII activity, Fv/Fm indicated the maximum photosynthetic efficiency of PSII and the potential maximum photoenergy conversion efficiency in plants. As reported in Figure 5, the Fv/Fm values for each treatment group decreased significantly compared to the control group after 66 h of incubation at the different *Z. bungeanum* leaf extract concentrations. Specifically, the Fv/Fm values reached their highest value in the control group, and the values under different concentration gradients of *Z. bungeanum* leaf extracts decreased by 33.11% (20 mg/L) and 54.60% (40 mg/L) compared to the control group at 66 h. Meanwhile, the Fv/Fm of *T. obliquus* decreased remarkably with the increased exposure time (Figure 5A). Similarly, the photosynthetic performance index (PIabs) reflected the plant’s overall photosynthetic performance and showed a declining trend during the test period. For example, *Z. bungeanum* leaf extract stress decreased the PIabs by 96.08% and 98.22% after exposure of *T. obliquus* to 20 and 40 mg/L for 66 h, respectively (Figure 5B).

**Figure 6:**
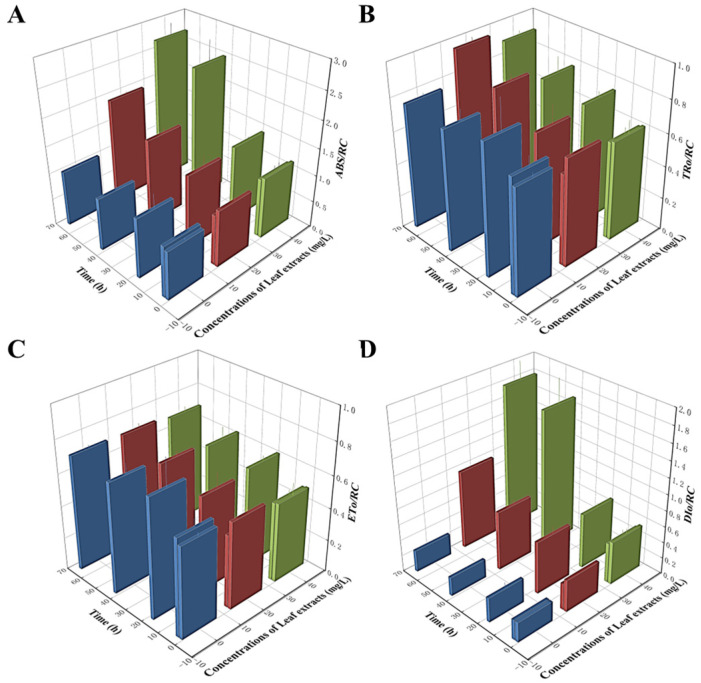
Variations in ABS/RC (**A**), TRo/RC (**B**), ETo/RC (**C**), and DIo/RC (**D**) of *T. obliquus* in response to exposure time to different *Z. bungeanum* leaf extract concentrations. Values represent the mean of three independent measurements (*n* = 3), and bars indicate SDs. All processes were biologically repeated in three independent and parallel experiments.

It is well-established that the PIabs value functions as an indicator of three essential photosynthetic processes: absorption (ABS), trapping (TR), and electron transport (ET). Therefore, we next examined the effect of *Z. bungeanum* leaf extracts on distributing light energy in the PSII reaction center. As shown in Figure 6, the variation tendency of four parameters related to light energy utilization was different. The ABS/RC values were higher in all treatment groups, with the highest ABS/RC value noted in the group treated at a concentration of 40 mg/L, which was 1.60-fold greater than that in the control group (Figure 6A). Additionally, the DIo/RC and TRo/RC values showed similar trends and were progressively greater than those of the control group (Figure 6B,D). However, the ETo/RC values showed gradual decreases with the increases in *Z. bungeanum* leaf extract concentrations, and these values were significantly lower (*p* < 0.05) than that of the control group. The treatment group treated at a concentration of 40 mg/L had the lowest ETo/RC value with a decrease of 3.10% compared to the control (Figure 6C). Our findings suggest that the excess light energy absorbed at the reaction centers of these algal cells is not utilized to enhance photosynthetic efficiency. Instead, this excess energy is dissipated as heat, potentially serving as a self-protection mechanism against environmental stressors.

**Figure 7:**
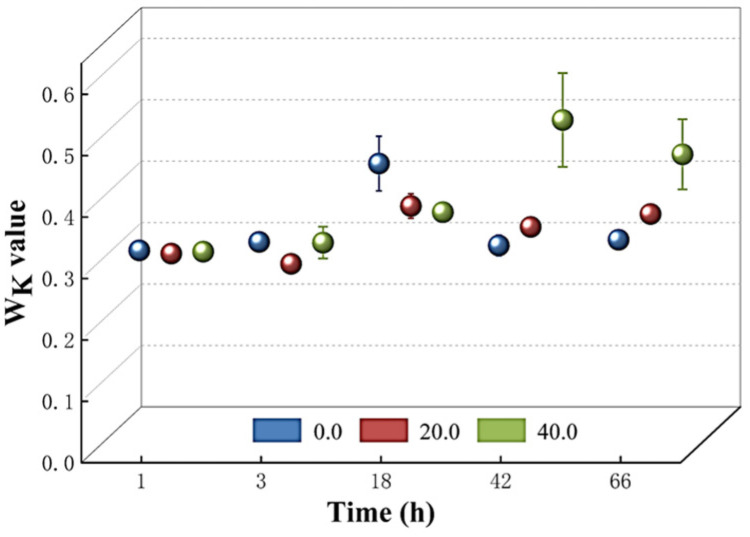
Comparison of W_k_ values of *T. obliquus* in response to exposure time to different *Z. bungeanum* leaf extract concentrations. Values represent the mean of three independent measurements (*n* = 3), and bars indicate SDs. All processes were biologically repeated in three independent and parallel experiments.

The structural integrity of the oxygen-evolving complex (OEC) on the donor side of the PSII reaction center was then assessed by measuring the W_k_ value. Figure 7 demonstrates that there was no significant increase in the W_k_ value among *T. obliquus* cells cultured with various doses of *Z. bungeanum* leaf extracts during the first 18 h. However, a rapid increase in the W_k_ value was observed with the rising dosages of *Z. bungeanum* leaf extract concentrations in the different treatment groups after 42 and 66 h of exposure. Administration of *Z. bungeanum* leaf extracts at concentrations of 20 mg/L and 40 mg/L resulted in W_k_ values that were 13.21% and 43.71% higher than that of the control. This suggests that the treatment with *Z. bungeanum* leaf extracts may disrupt the structure of the OEC.

**Figure 8:**
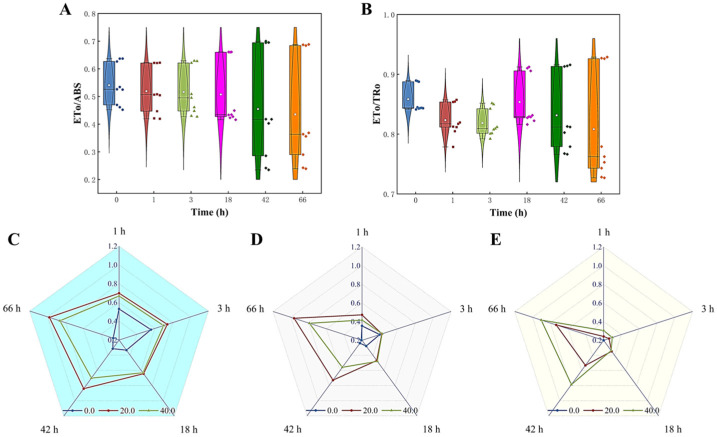
Comparison of five parameters related to electron transport of the fluorescence induction kinetics curves of *T. obliquus* in response to exposure time to different *Z. bungeanum* leaf extract concentrations. (**A**) ETo/ABS, (**B**) ETo/TRo, (**C**) V_J_, (**D**) Mo, and (**E**) Sm. All processes were biologically repeated in three independent and parallel experiments.

The effect of *Z. bungeanum* leaf extracts on the efficiency of electron transfer at the receptor side of the PSII reaction center of *T. obliquus* was evaluated by measuring the ETo/ABS and ETo/TRo values. Figure 8A,B demonstrate a steady and significant decrease in the ETo/ABS and ETo/TRo values with increasing treatment concentration of *Z. bungeanum* leaf extract. At a dose of 40 mg/L *Z. bungeanum* leaf extract, the experimental group had the lowest ETo/ABS and ETo/TRo values. Specifically, the ETo/ABS and ETo/TRo values were reduced by 62.54% and 17.48%, respectively, compared to the control group after 66 h. In addition, the Mo, Sm, and V_J_ values exhibited a positive response to *Z. bungeanum* leaf extract. These results indicated that exposure of *T. obliquus* to the *Z. bungeanum* leaf extract resulted in a significant increase (*p* < 0.05) in these values across all tested groups (Figure 8C–E).

### 3.4. Effect of Different Z. bungeanum Leaf Extract Concentrations on the Total Antioxidant Activity in T. obliquus

**Figure 9:**
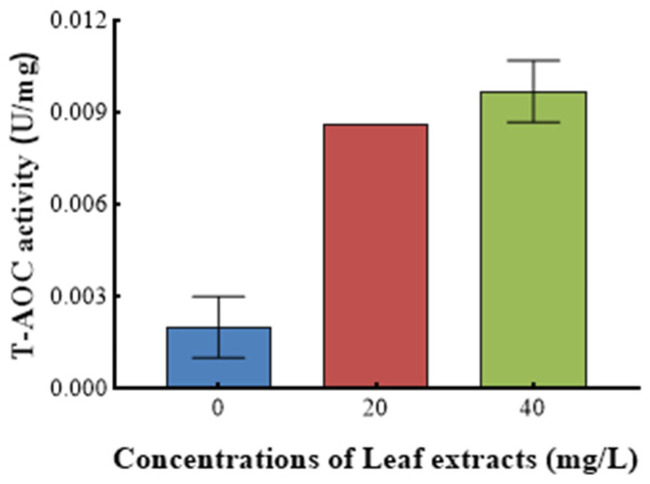
Effect of different *Z. bungeanum* leaf extract concentrations on the T-AOC activity of *T. obliquus* at 66 h. Values represent the mean of three independent measurements (*n* = 3), and bars indicate SDs. All processes were biologically repeated in three independent and parallel experiments.

Figure 9 shows the effect of *Z. bungeanum* leaf extract on the total antioxidant activity (T-AOC) of *T. obliquus*. A statistically significant variation in T-AOC (*p* < 0.05) was observed among *T. obliquus* cells grown with varying doses of *Z. bungeanum* leaf extracts. The T-AOC exhibited a positive correlation with increases in concentrations of *Z. bungeanum* leaf extract. Treatment with *Z. bungeanum* leaf extracts at doses of 20 mg/L and 40 mg/L resulted in T-AOC values that were 3.5 times higher and 4.0 times higher than the control, respectively (Figure 9).

## 4. Discussion

Due to the antioxidant, antitumor, anti-inflammatory, antimicrobial, and insecticidal activity of *Z. bungeanum*, extensive research has led to the isolation and identification of its active ingredients, with flavonoids being reported as the most important active constituents. The leaves of *Z. bungeanum* contain functional components similar to those found in the plant itself and are primarily used in nutrition, food, and medicine given their abundant flavonoid content and associated health benefits. These leaves contain antioxidant flavonoids that are effective in scavenging free radicals, but the antimicrobial properties of flavonoid components from *Z. bungeanum* leaves have received little attention. We optimized the ultrasonic-assisted extraction method for TFs from *Z. bungeanum* leaves. Subsequently, we investigated the effects of different doses of *Z. bungeanum* leaf extracts on the growth of *T. obliquus*.

### 4.1. Optimization of Total Flavonoid Extraction from Z. bungeanum Leaf

Various factors, such as ultrasonic power, extraction solvent, extraction time, extraction temperature, and LSR, have been proven to have an effect on the extraction process [20,21]. In this study, a single-factor approach and the response surface technique were implemented to examine the effect of the aforementioned variables on extracting TFs from *Z. bungeanum* leaf. A suitable range of five factors influencing the extraction rate of TFs was identified and excluded. Undoubtedly, the magnitude of ultrasonic power was really significant in augmenting the total production of flavonoids. Evidence shows that the extraction efficiency of TFs improved with higher ultrasonic power ranging from 300 W to 600 W (Figure S2). The results indicated that an increase in the ultrasonic power facilitated enhanced penetration of solvents into cells and increased passage of target chemicals through the cell wall. Meanwhile, an increase in ultrasound power will improve the molecular vibration speed and strengthen intermolecular interactions. The above results provided strong evidence that higher ultrasound power could boost the total flavonoid yield.

The influence of the LSR on the rate of flavonoid extraction is shown in Figure 3A. The results indicated that the production of flavonoids from *Z. bungeanum* leaves exhibited a progressive increase as the LSR rose. However, a decline in yield was found when the LSR exceeded 20:1 mL/g. The optimization of the LSR is crucial as a larger ratio might lead to excessive use of extract solvent, while a lower ratio can result in inadequate extraction of flavonoids. We thus examined how the concentration of ethanol affects the extraction yield of TFs. The extraction efficiency was significantly improved as the ethanol concentration increases from 20% to 80%, but thereafter declined with greater ethanol concentrations (Figure 3B). This may be because the higher concentrations of ethanol have better flavonoid solubility due to its strong cell penetration properties. Nevertheless, when the ethanol concentration exceeds a certain threshold, the solubilization of TFs will be influenced by the absorption of highly lipophilic components and the elevation in certain alcohol-soluble pigments [40]. Consequently, the highest flavonoid extraction yield was achieved when the ethanol concentration reached the correct ratio, which is consistent with previous research findings [41]. In addition, the duration of ultrasonic treatment had a significant consequence on the overall extraction efficiency of TFs (Figure 3C). As the extraction time increased, the highest yield of TFs first increased and then decreased, peaking at 20 min, indicating that the degree of cell fragmentation would subsequently increase as the ultrasonic time increased. Nevertheless, an overlong extraction time would cause thermal and mechanical effects that would damage the flavonoid structure [42]. Therefore, it was essential to determine the optimal duration of ultrasonic processing for flavonoid extraction. We aimed to investigate the effect of ultrasonic temperature on the rate of total flavonoid extraction while keeping other extraction parameters constant. A higher temperature initially increases the dissolution of flavonoids. From Figure 3D, it can be seen that the flavonoid yield reached its maximum value under an ultrasonic temperature of 80 °C. This was because molecules of flavonoid compounds had increased thermal kinetic energy and accelerated diffusion rates with the increase in ultrasonic temperature, thus increasing the solubility of flavonoid compounds in the solvent and subsequently improving the flavonoid yield [43].

Using a three-factor, three-level BBD model, the extraction process was improved based on the results of prior single-factor simulations. The measured values revealed that the flavonoid production varied between 1.397 and 6.989. The optimal production was obtained with an LSR of 20:1 mL/g, an ultrasonic duration of 20 min, and an ethanol concentration of 80% (Table 1). The ANOVA findings from the BBD experiment are also shown in Table 2. The correlation coefficient (R^2^) and the F value were 0.9551 and 16.53, respectively. The statistical significance (*p* < 0.0001) of the model and non-significance of lack-of-fit (*p* = 0.0580) indicate that the second-order polynomial model is appropriate for predicting the response values. Furthermore, a strong linear connection between ethanol concentration and the rate of ultrasonic extraction of flavonoids was found. This was confirmed by the *p*-value of A being less than 0.05 and the *p*-values of A^2^ and B^2^ being less than 0.05. This study demonstrates that the concentration of ethanol has a significant curvilinear effect on the pace of ultrasonic extraction. Therefore, the *p*-values for AB, AC, and BC were all more than 0.05, indicating that the interaction effects among different factors on flavonoid production were not significant. The above results provided evidence that there was no simple linear or quadratic correlation among the independent variables. Following the analysis of the F-value in the ANOVA findings, it is hypothesized that the concentration of ethanol had the most significant influence on flavonoid yield. Ultrasound duration followed closely after, while the impact of the LSR on the production of flavonoids was the least significant (Table 2).

Subsequently, the interaction effects between any two variables affecting flavonoid yield were then further analyzed and evaluated using response surface plots and contour plots. In response surface plots, a higher gradient of the response surface signifies a more significant effect of the factor on the response value, therefore illustrating a greater degree of sensitivity of the flavonoid extraction rate to variations in that component. Analysis of the contour plot supported the conclusions of the response surface plots. Specifically, curves closer to the center denoted larger corresponding response values. Additionally, a contour shape approximating a circle suggested a weaker interaction between the two independent variables. When the shape of the response surface approximated an ellipse, this suggested that the corresponding response value increased as the curve approached the center [44]. Furthermore, a convex response surface indicated that the range of variable was appropriately set [45]. In our study, the circular contour plots revealed that the interactions between ethanol concentration and ultrasonic time, ethanol concentration and LSR, and ultrasonic time and LSR were not significant.

### 4.2. Evaluation of the Antimicrobial Properties of Z. bungeanum Leaf Extracts

Extracts of *Z. bungeanum* leaves were synthesized for successive toxicological studies based on the response surface methodology. The antimicrobial effects of flavonoid components from *Z. bungeanum* leaves on the suppression of *T. obliquus* were previously unknown, thereby making it a novel biotechnological algicide. In this study, we examined the effect of *Z. bungeanum* leaf extracts on the cell density of *T. obliquus* by administering varying concentrations of the extracts (Figure 1). The results indicated that the treatments with different concentrations of *Z. bungeanum* leaf extracts significantly inhibited the growth of *T. obliquus* in a concentration-dependent manner, and our results were similar to a previous study, in which crude extracts of *Zostera marina* negatively affected the growth of *Alexandrium catenella* [46]. Additionally, the cells exhibited an uneven distribution and aggregation in certain dense regions when exposed to *Z. bungeanum* leaf extracts, suggesting a potential maladaptive response to stress conditions. Overall, *Z. bungeanum* leaf extracts have demonstrated growth suppression of *T. obliquus* in our study, which is consistent with other studies showing the inhibitory effect of natural flavonoids or flavonoid-containing plant extracts on the growth of *Microcystis aeruginosa* [46,47].

PSII is integral to photosynthesis, facilitating the absorption, transfer, and conversion of light energy. Changes in PSII reaction center parameters under stress conditions serve as indicators of a plant’s response to environmental adversity [48]. Chlorophyll fluorescence induction kinetic curves are used to assess the impact of contaminants on the electron transport chain of PSII in microalgae, with the JIP test providing a quantitative measure of PSII performance [49,50]. Our study indicated that the shapes of the fluorescence induction curves exhibited more pronounced alterations across different concentration gradients of *Z. bungeanum* leaf extracts, demonstrating that the photosynthetic apparatus may respond to *Z. bungeanum* leaf extracts by closing PSII reaction centers.

The photosynthetic performance of *Z. bungeanum* leaf extract-treated *T. obliquus* was then evaluated using the JIP test. The results indicated that the *Z. bungeanum* leaf extracts had a significant consequence on the photosynthetic capacity of *T. obliquus*. Higher concentrations of *Z. bungeanum* leaf extract, ranging from 20 to 40 mg/L, resulted in greater inhibitory effects on the effective quantum yield (Fv/Fm) and photosynthetic performance index (PIabs), indicating that the light energy conversion efficiency of *T. obliquus* was inhibited under all *Z. bungeanum* leaf extract treatment conditions. Similar results have also been reported in a previous paper [51]. Furthermore, our finding that *Z. bungeanum* leaf extract treatment caused a significant increase in the ABS/RC and DIo/RC of *T. obliquus* revealed that the addition of *Z. bungeanum* leaf extracts inactivated the reaction centers, leading to an increase in light absorption capacity and heat dissipation energy of the PSII unit reaction centers. However, the energy utilized for electron transfer decreases due to the decrease in ETo/RC, leading to a decrease in the photosynthetic performance index. The underlying interactions between *Z. bungeanum* leaf extracts and *T. obliquus* likely include the disruption of electron transport in the PSII reaction center. This disruption occurs through the impairment of the secondary electron acceptor (Q_B_) complex function and a reduction in the effective quantum yield, ultimately resulting in compromised photosynthesis [52]. Altogether, an increase in the extract concentration resulted in a decrease in photosynthetic efficiency, and the potential inhibitory mechanisms of *Z. bungeanum* leaf extracts on *T. obliquus* may involve interruption of the electron transfer chain, photosynthetic rate, and membrane integrity [51].

Finally, we investigated the effects of *Z. bungeanum* leaf extracts on the T-AOC of *T. obliquus*. Algae are capable of generating reactive oxygen species (ROS) under adverse conditions that can disrupt the oxidative and non-oxidative balance within algal cells. These ROS are generated via the electron transport chain, and their enhanced generation may result in oxidative damage [53,54,55]. In our study, increasing the concentration of *Z. bungeanum* leaf extract from 20 to 40 mg/L led to an increase in T-AOC, suggesting that the elevation in T-AOC served as a primary biomarker for the intensity of oxidative stress. Meanwhile, an increase in extract concentration resulted in a decrease in the electron transport rate, leading to the accumulation of electrons and the generation of harmful ROS [56].

### 4.3. Eco-Friendly Algicidal Potential of Z. bungeanum Leaf Extracts

Plant-based or synthetic algaecides are often employed to manage harmful algal blooms, which can have detrimental effects on water quality, aquatic life, and human health. One study explored the use of an integrated metabolomics platform to investigate the effects of three commonly used algaecides—copper sulfate (CuSO_4_), hydrogen peroxide (H_2_O_2_), and sodium carbonate peroxide (SCP)—on the cyanobacterium *M. aeruginosa*. This study found that CuSO_4_ rapidly killed algae cells but caused secondary contamination through the release of microcystins, while H_2_O_2_ and SCP had different metabolic impacts on the algae [57]. Moreover, the impact of algaecides on *Scenedesmus* has also been studied. For instance, the effects of acetochlor, a synthetic herbicide, on the interaction between *Scenedesmus* and *Microcystis* were examined. This study revealed that acetochlor suppressed *Scenedesmus* growth, placing it at a competitive disadvantage in environments with herbicide pollution [58]. Additionally, the sensitivity of *Scenedesmus obliquus* to atrazine, another synthetic herbicide, was assessed, showing that *Scenedesmus* had lower growth rates in mixed cultures with *Microcystis* under atrazine exposure [59]. While synthetic algaecides like atrazine and acetochlor can be effective, they may also pose risks of secondary contamination and environmental harm. Plant-based algaecides, particularly those derived from seaweeds and macrophytes, offer a promising alternative with potentially fewer adverse effects on the ecosystem. One study highlighted the potential of plant-based algaecides, such as those derived from macrophyte metabolites, which showed effectiveness in reducing cyanobacterial biomass and microcystin concentrations in experimental microcosms [60]. Furthermore, the use of natural compounds from seaweeds has been proposed as an alternative to synthetic algaecides. Seaweeds contain bioactive compounds with antimicroalgae properties, which can be used to control harmful algae in aquatic systems [61]. This approach is considered environmentally friendly and sustainable compared to traditional chemical treatments.

It is worth noting that this study explores, for the first time, the use of *Z. bungeanum* leaf extracts as a biological algicide and investigates its impact on algal physiology. The results demonstrated that an elevation in extract concentrations resulted in an inhibition of algal cell density, and the extracts significantly inhibited the photosynthetic efficiency and activated the antioxidant system of algal cells. These findings of this study enhance our comprehension of the allelopathic mechanisms underlying *Z. bungeanum* leaf extract and provide important theoretical support for algal bloom control strategies. While the current research was conducted under controlled experimental conditions, further investigations are needed to assess its effects on mixed algal communities in natural aquatic environments. Additionally, developing sustained-release microsphere formulations incorporating *Z. bungeanum* leaf extract and carrier materials could enable continuous release of allelochemicals, thereby achieving prolonged algal suppression. Subsequent field trials should prioritize comprehensive evaluations of both immediate inhibitory effects and potential long-term ecological consequences. Despite these research gaps, the demonstrated algicidal properties of *Z. bungeanum* leaf extract highlight its potential as a promising novel algicidal agent for the sustainable management of harmful algal blooms. Future studies should focus on optimizing application protocols and conducting systematic environmental risk assessments to facilitate practical implementation.

## 5. Conclusions

In this work, the ultrasonic-assisted extraction procedure to obtain TFs from *Z. bungeanum* leaves was optimized using response surface methodology. Subsequently, the harmful effects of different doses of *Z. bungeanum* leaf extract on fresh microalgae *T. obliquus* were investigated. The analysis showed that the extraction yield of TFs from *Z. bungeanum* leaves reached 7.0% under the optimized circumstances. Furthermore, an elevation in extract concentrations resulted in an inhibition of algal cell density, and the extracts significantly inhibited the photosynthetic efficiency and activated the antioxidant system of algal cells, demonstrating that *Z. bungeanum* leaf extracts could be used in the development of potentially effective biological algicides. This study provides data to support the development of algicides and realizes the resource application of *Z. bungeanum* leaf waste, achieving a synergistic outcome of both economic and ecological benefits.

## Figures and Tables

**Table 1:** Box–Behnken design (BBD) for the independent variables and corresponding response values.

Run Order	Ethanol Concentration	Ultrasonic Time	Liquid-to-Solid Ratio	Response Value
1	80	20	20	6.989
2	100	20	25	2.400
3	100	30	20	1.397
4	80	20	20	6.847
5	80	20	20	6.845
6	80	30	15	5.242
7	80	10	25	5.003
8	100	10	20	2.713
9	80	20	15	5.598
10	80	10	20	6.154
11	60	30	20	4.315
12	60	30	20	2.118
13	100	20	15	3.140
14	80	20	20	6.818
15	60	20	15	3.989
16	60	20	25	4.842
17	80	30	25	5.667

**Table 2:** ANOVA for the regression quadratic model equation of BBD.

Source	Sum of Squares	df	Mean Square	F-Value	*p*-Value	
**Model**	50.71	9	5.63	16.53	0.0006	Significant
A-Ethanol concentration	3.94	1	3.94	11.56	0.0114	*
B-Ultrasonic time	1.28	1	1.28	3.77	0.0934	
C-Liquid-to-solid ratio	0.0004	1	0.0004	0.0012	0.9734	
AB	0.1940	1	0.1940	0.5694	0.4751	
AC	0.6344	1	0.6344	1.86	0.2147	
BC	0.2601	1	0.2601	0.7632	0.4113	
A2	36.39	1	36.39	106.77	<0.0001	*
B2	5.62	1	5.62	16.48	0.0048	*
C2	0.1652	1	0.1652	0.4846	0.5088	
**Residual**	2.39	7	0.3408			
Lack of Fit	1.95	3	0.6507	6.00	0.0580	Not significant
Pure Error	0.4335	4				
**Cor Total**	53.10	16				

* indicated a significant difference at the 0.05 level (*p* < 0.05).

## Data Availability

The original contributions presented in this study are included in the article/Supplementary Material. Further inquiries can be directed to the corresponding authors.

## Supplementary Materials

The following supporting information can be downloaded at: https://www.mdpi.com/article/10.3390/microorganisms13040760/s1, Figure S1. The relationship between concentrations of rutin and OD_510_ values. Values represent the mean of three independent measurements (*n* = 3). All processes were biologically repeated in three independent and parallel experiments. Figure S2. The effects of ultrasound power on the extraction performance of *Z. bungeanum* leaves. Values represent the mean of three independent measurements (*n* = 3) and bars indicate SD. All processes were biologically repeated in three independent and parallel experiments. Figure S3. Perturbation plots for the three factors affecting flavonoids yield on the basis of the regression equation. (A) Ethanol concentration, (B) Ultrasonic time, and (C) Liquid-solid ratio. Figure S4. Effect of *Z. bungeanum* leaf extracts concentration treatments on the chlorophyll a fluorescence induction kinetic curves of *T. obliquus* at 1 h (A), 3 h (B), 18 h (C), 42 h (D), and 66 h (E). Table S1. Actual and coded levels of the factors of Box-Behnken design (BBD). Table S2. The flavonoids compounds detected in *Z. bungeanum* leaf extracts using the non-targeted metabolomics analysis. Table S3. The optimal flavonoid content in samples 1, samples 2, and samples 3 by the single factor tests.
