# Supplementary material for: Eco-Friendly Algicidal Potential of Zanthoxylum bungeanum Leaf Extracts: Extraction Optimization and Impact on Algal Growth
Source: Microorganisms. 2025 Mar 27;13(4):760. doi: 10.3390/microorganisms13040760 (PMC12029162; doi:10.3390/microorganisms13040760)
Supplement: Supplementary file 1 [file microorganisms-13-00760-s001.zip › microorganisms-3535908-supplementary.pdf]

Supplementary Information

Eco-Friendly Algicidal Potential of *Zanthoxylum bungeanum* Leaf Extracts: Extraction Optimization and Impact on Algal Growth

Jie Cheng [1], Long Tan [1], Yaxin Han [1], Mengya Hou [1], Zhenxia Zhu [1], Xiu Zhang [1], Qing Guo [1], Kaidian Zhang [2], Jiashun Li [2,*], Yang Zhang [1], Chaobo Zhang [1,*]

[1] State Key Laboratory of Macromolecular Drugs and Large-Scale Preparation, School of Pharmaceutical Sciences and Food Engineering, Liaocheng University, Liaocheng 252000, China;

[2] State Key Laboratory of Marine Resource Utilization in the South China Sea, Hainan University, Haikou 570100, China.

* Corresponding authors:

jiashun.li@hainanu.edu.cn (Jiashun Li); zhangchaobo@lcu.edu.cn (Chaobo Zhang)

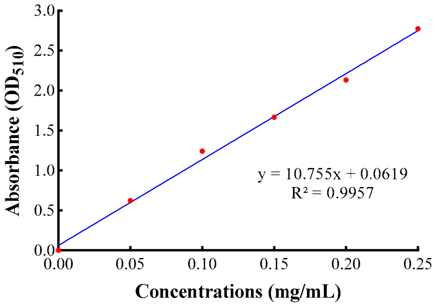

Figure S1 The relationship between concentrations of rutin and OD_{510} values. Values represent the mean of three independent measurements (n = 3). All processes were biologically repeated in three independent and parallel experiments.

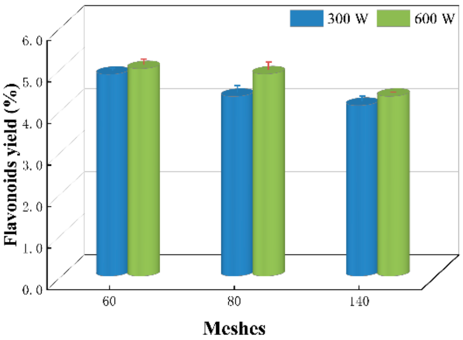

Figure S2 The effects of ultrasound power on the extraction performance of *Z. bungeanum* leaves. Values represent the mean of three independent measurements (n = 3) and bars indicate SD. All processes were biologically repeated in three independent and parallel experiments.

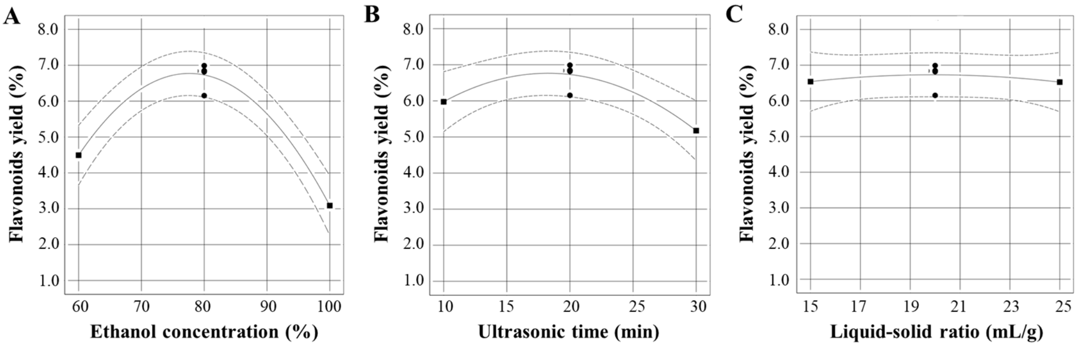

Figure S3 Perturbation plots for the three factors affecting flavonoids yield on the basis of the regression equation. (A) Ethanol concentration, (B) Ultrasonic time, and (C) Liquid-solid ratio.

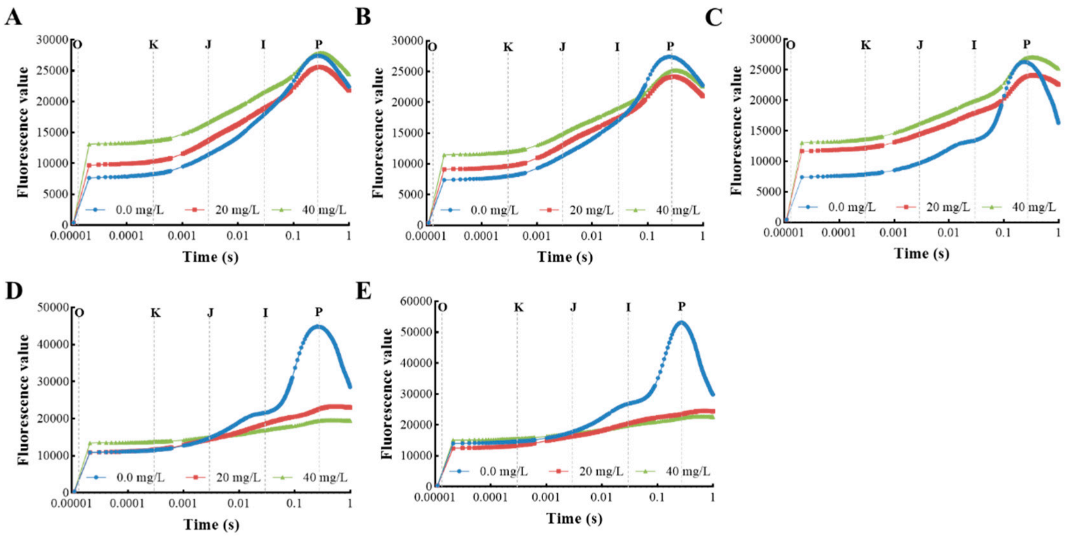

Figure S4 Effect of *Z. bungeanum* leaf extracts concentration treatments on the chlorophyll a fluorescence induction kinetic curves of *T. obliquus* at 1 h (A), 3 h (B), 18 h (C), 42 h (D), and 66 h (E).

Table S1 Actual and coded levels of the factors of Box–Behnken design (BBD)

Factor	Name	Units	Coded Low	Coded High	Mean	Std.Dew
**A**	Ethanol concentration	%	-1→60.0	+1→100.0	80	14.14
**B**	Ultrasonic time	min	-1→10.0	+1→30.0	20	7.07
**C**	Liquid-solid ratio	mL/g	-1→15.0	+1→25.0	20	3.54

Table S2 The flavonoids compounds detected in *Z. bungeanum* leaf extracts using the non-targeted metabolomics analysis

Peak No.	Subclass	RT*	Compounds	Area
1	Flavones	4.09	Naringenin-7-O--D-Glucuronide	5066883.25
2	Flavones	3.90	3''-O-L-Rhamnopyranosylastragalin	2323343.76
3	Flavones	3.33	Butrin	872845.43
4	Flavones	2.09	4'-Hydroxyflavone	645791.49
5	Flavones	4.68	[6-[2-(3,4-Dihydroxyphenyl)-5,7-dihydroxy-4-oxochromen-3-yl]oxy-3,4,5-trihydroxyoxan-2-yl]methyl acetate	623723.50
6	Flavones	3.69	Quercetin 3-(2-glucosylrhamnoside)	234646.72
7	Flavones	3.77	5,7,2',4'-Tetrahydroxy-3-geranylflavone	154435.22
8	Flavones	4.21	Gambiriin C	152295.46
9	Flavones	4.48	Troxerutin	112929.23
10	Flavones	4.37	6-(3,3-DMA)chrysin	102251.89
11	Flavones	4.09	Vitexin 2''-O-rhamnoside	87164.37
12	Flavones	3.43	2-(3,4-dihydroxyphenyl)-5,7-dihydroxy-6-[(2S,3R,4R,5S,6R)-3,4,5-trihydroxy-6-(hydroxymethyl)oxan-2-yl]-8-[(2S,3R,4S,5S)-3,4,5-trihydroxyoxan-2-yl]chromen-4-one	71186.61
13	Flavones	3.43	Vincetoxicoside A	68590.81
14	Flavones	4.16	Quercetin 3-xylosyl-(1->6)-glucoside	61982.97

15	Flavones	3.77	6-Geranylchrysin	36805.93
16	Flavones	3.85	Acacetin diacetate	36178.32
17	Flavones	3.28	Robinetinidol-(4alpha->8)-catechin-(6->4alpha)-robinetinidol	34221.16
18	Flavones	4.79	Nateglinide	31435.76
19	Flavones	6.20	4'-Methoxyflavone	31368.67
20	Flavones	6.35	Diosmetin	25507.6
21	Flavones	3.46	Spinosin	23485.08
22	Flavones	4.39	Kandelin A-1	21122.31
23	Flavones	4.81	Nepitrin	17091.38
24	Flavones	2.62	2''-p-Coumarylastragalin	14587.69
25	Flavones	6.18	Acerosin	12338.61
26	Flavones	5.81	5,7,3',4'-Tetrahydroxy-6,8-dimethoxyflavone	12079.7
27	Flavones	3.71	Typhaneoside	11072.53
28	Flavones	1.14	Isorhamnetin 3-O-alpha-rhamnopyranosyl-(1-2)-beta-galactopyranoside	10073.12
29	Flavones	4.59	Kaempferol-3-Rhamnoside-4''-Rhamnoside-7-Rhamnoside	9414.41
30	Flavones	4.49	Laricitrin 3-glucoside	7780.92
31	Flavones	5.18	Laricitrin 3-galactoside	5351.16
32	Flavones	4.84	(2S,3R,4S,5S,6R)-2-[2-[1-hydroxy-3-(4-hydroxy-3-methoxyphenyl)propan-2-yl]-4-[(E)-3-hydroxyprop-1-enyl]-6-	5303.00

			methoxyphenoxy]-6-(hydroxymethyl)oxane-3,4,5-triol	
33	Flavones	5.04	7,3',4',5'-Tetrahydroxyflavone	4585.46
34	Flavones	5.01	Gossypetin 8-rhamnoside	3179.43
35	Flavones	4.90	Dactilin	2306.29
36	Flavones	1.86	5'-Prenylhomoeriodictyol	920.61
37	Flavones	3.35	4'-Hydroxyflavanone	267.4
38	Flavonols	4.42	Rutin	11011075.62
39	Flavonols	4.56	Hyperoside	4318166.41
40	Flavonols	6.36	3,7-Di-O-methylquercetin	1387527.87
41	Flavonols	4.64	2-(3,4-dihydroxyphenyl)-5-hydroxy-3,7-bis[[(2S,3R,4R,5R,6S)-3,4,5-trihydroxy-6-methyloxan-2-yl]oxy]chromen-4-one	950961.32
42	Flavonols	4.56	Myricitrin	625168.59
43	Flavonols	4.73	3-[(2R,3R,4R,5S)-3,4-dihydroxy-5-(hydroxymethyl)oxolan-2-yl]oxy-2-(3,4-dihydroxyphenyl)-5,7-dihydroxychromen-4-one	148694.54
44	Flavonols	4.68	Quercetin 3-O-(6''-acetyl-glucoside)	141501.12
45	Flavonols	3.51	kaempferol 3-O-beta-D-glucopyranosyl-7-O-alpha-L-rhamnopyranoside	89181.89
46	Flavonols	0.81	3-Hydroxyflavone	82474.50
47	Flavonols	8.81	Kaempferol-7-rhamnoside	66330.88
48	Flavonols	5.42	Quercetin	33512.96

49	Flavonols	3.51	kaempferol 3-O-[alpha-L-rhamnopyranosyl(1->2)-beta-D-glucopyranosyl]-7-O-alpha-L-rhamnopyranoside	20168.73
50	Flavonols	5.78	Isorhamnetin	12877.15
51	Flavonols	0.84	Flavonol 3-O-rutinoside	2408.7
52	Flavonols	4.94	kaempferol 7-O-glucoside	992.07
53	Flavonols	5.72	Kaempferol	826.02
54	Flavonols	3.10	Syringetin-3-o-glucoside	782.16
55	Chalcones	4.52	Naringenin-4'-o-beta-d-glucuronide	365713.54
56	Chalcones	3.21	Licochalcone a	57062.25
57	Chalcones	3.78	Neohesperidin dihydrochalcone	50933.53
58	Chalcones	2.29	Xanthohumol	49819.78
59	Chalcones	6.08	Flavokawain b	1232.21
60	Chalcones	2.35	Isobavachalcone	864.56
61	Flavanones	2.50	Glabranin	448347.55
62	Flavanones	3.85	Farrerol	241908.19
63	Flavanones	2.91	Lonchocarpol A	58754.42
64	Flavanones	2.88	Didymin	43644.59
65	Flavanones	4.80	Hesperetin	13974.94
66	Flavanones	2.41	Liquiritin	656.79

67	Anthocyanidins	2.93	Pelargonidin 3-(6-p-coumaroyl)glucoside	5446.69
68	Anthocyanidins	4.92	Cyanidin 3-arabinoside cation	856.28
69	Anthocyanidins	0.95	Delphinidin 3-sambubioside	409.5
70	Flavanols	3.31	Cianidanol	306847.53
71	Flavanols	1.01	Epicatechin-3'-glucuronide	56327.58
72	Flavanols	4.68	Gallocatechin gallate	37343.71
73	Flavanols	3.77	(-)-Epicatechin	16842.73
74	Flavanols	3.47	(-)-Epicatechin gallate	2259.07
75	Flavanols	3.99	(2S,3S)-2-(3,4-dihydroxyphenyl)-8-[(2R,3R,4R)-2-(3,4-dihydroxyphenyl)-3,5,7-trihydroxy-chroman-4-yl]chromane-3,5,7-triol	1920.7
76	Biflavones	4.29	Isoginkgetin	36121.52
77	Isoflavones	4.51	Sophoricoside	13581484.33
78	Isoflavones	4.15	Biochanin A 7-O-(6-O-malonyl-beta-D-glucoside)	142989.79
79	Isoflavones	5.02	Genistin	56457.4
80	Isoflavones	4.90	Irigenin, dibenzyl ether	52990.41
81	Isoflavones	3.07	7-hydroxy-3-(2-methoxyphenyl)-4H-chromen-4-one	51140.33
82	Isoflavones	1.75	Ipriflavone	10650.18
83	Other Flavonoids	6.06	Garcinone D	290178.38
84	Other Flavonoids	3.16	Vestitol	201980.54

85	Other Flavonoids	2.88	Irisxanthone	114037.8
86	Other Flavonoids	0.80	alpha-Mangostin	49940.25
87	Other Flavonoids	3.35	(E)-4-(3,7-Dimethyl-2,6-octadienyl)-1,3,5-trihydroxyxanthone	26820.19
88	Other Flavonoids	4.66	Mangiferin	7962.37
89	Other Flavonoids	2.38	Calabaxanthone	6188.00
90	Other Flavonoids	3.13	1,6-Dihydroxy-3,7-dimethoxy-2-(3-methyl-2-butenyl)-8-(2-oxo-3-methyl-3-butenyl)-xanthone	2478.37

*RT: Retention Time (min).

Table S3 The optimal flavonoid content in samples 1, samples 2, and samples 3 by the single factor tests

Samples	Mass before sifting / g	Mass after sifting / g	Flavonoids yield / %	Flavonoids content / g
1	100.00	68.60	5.204	3.570
2	100.00	54.70	6.720	3.676
3	100.00	26.40	5.269	1.391
